# Helminth Infections in Dairy Sheep Found in an Extensive Countrywide Study in Greece and Potential Predictors for Their Presence in Faecal Samples

**DOI:** 10.3390/microorganisms11030571

**Published:** 2023-02-24

**Authors:** Daphne T. Lianou, Konstantinos V. Arsenopoulos, Charalambia K. Michael, Vasia S. Mavrogianni, Elias Papadopoulos, George C. Fthenakis

**Affiliations:** 1 Veterinary Faculty, University of Thessaly, 43100 Karditsa, Greece; 2 Laboratory of Parasitology and Parasitic Diseases, School of Veterinary Medicine, Faculty of Health Sciences, Aristotle University of Thessaloniki, 54124 Thessaloniki, Greece

**Keywords:** diarrhoea, *Dicrocoelium dendriticum*, epg counts, lungworms, nematode, parasite, predictor, sheep, trematode, Trichostrongylidae

## Abstract

The aims of the present study were: (a) to describe the prevalence of helminth infections from pooled faecal samples from sheep flocks across Greece and (b) to evaluate flock-related factors potentially associated with the presence of these infections in the flocks. An extensive countrywide study was performed on 325 sheep farms throughout Greece; faecal samples were collected from ewes and processed for the identification of helminth parasites. Helminths were detected in samples from 92.9% of flocks; these included *Dicrocoelium dendriticum* (16.7% of flocks), *Fasciola hepatica* (0.6%), *Paramphistomum cervi* (2.2%), *Moniezia* spp. (18.8%), Trichostrongylidae (85.5%), *Nematodirus* spp. (18.8%), *Strongyloides papillosus* (7.1%), *Trichuris* spp. (20.0%) and lungworms (17.8%). Mean Trichostrongylidae counts across all flocks in the study were 215 epg. Specifically, for Trichostrongylidae, there were differences between flocks that had or had not received anthelmintics during the two months prior to sampling, as well as between flocks located in various areas of the country. In multivariable analyses, for the outcome ‘high (>300) epg counts in faecal samples’, the month into the lactation period at sampling and the application of reproductive control practices on the farm emerged as significant factors; for the outcome ‘high proportion (>63%) of *Teladorsagia* spp. in faecal samples’, the availability of straw bedding emerged as a significant factor; and for the outcome ‘high proportion (>63%) of *Haemonchus contortus* in faecal samples’, the age of the farmer emerged as a significant factor. For the outcome ‘presence of *Trichuris* spp. in faecal samples’, the provision of finished feed (concentrate) to animals emerged as a significant factor, whilst, finally, for the outcomes ‘presence of *D. dendriticum* in faecal samples’ and ‘presence of lungworms in faecal samples,’ no significant factors emerged.

## 1. Introduction

Gastrointestinal helminth infections are well-documented as major contributors to the reduced productivity of affected animals [1,2]. Their adverse effects on sheep (*Ovis aries*, Linnaeus 1758) health and production have been repeatedly presented.

In dairy sheep production systems, previous publications have reported that gastrointestinal nematode parasitism can cause from 10 to 15% reduction in the milk yield of affected ewes [3,4]. Moreover, helminth infections have been found to be associated with reduced fat and protein contents in milk [4,5]. Additionally, these infections have been considered as potential risk factors for mastitis in ewes, as found in field (for trematodes [6] and nematodes [7]) or in experimental [8] studies [9].

Greece has a high number of sheep, an estimated 8,400,000 [10], which account for approximately 6.5% of the total number in Europe [11]. Sheep farming in Greece aims mainly for dairy production. Milk production amounts to 645,000 tonnes annually [11,12] and those quantities exceed the milk production of cattle in this country [13]. Over 97% of the total milk produced from sheep is used for the manufacturing of dairy products.

Therefore, there is scope for monitoring the situation regarding helminth infections in sheep in this country, given that the respective infections can affect the quantity and quality of milk produced on farms. This is of greater significance when considering the presence of anthelmintic resistance in this country [14], which may complicate the health management of flocks.

The aims of the present study were: (a) to describe the prevalence of helminth infections from pooled faecal samples from sheep flocks across Greece and (b) to evaluate flock-related factors potentially associated with the presence of these infections in the flocks.

## 2. Materials and Methods

### 2.1. Sheep Flocks and the Collection of Samples and Information

**Figure 1:**
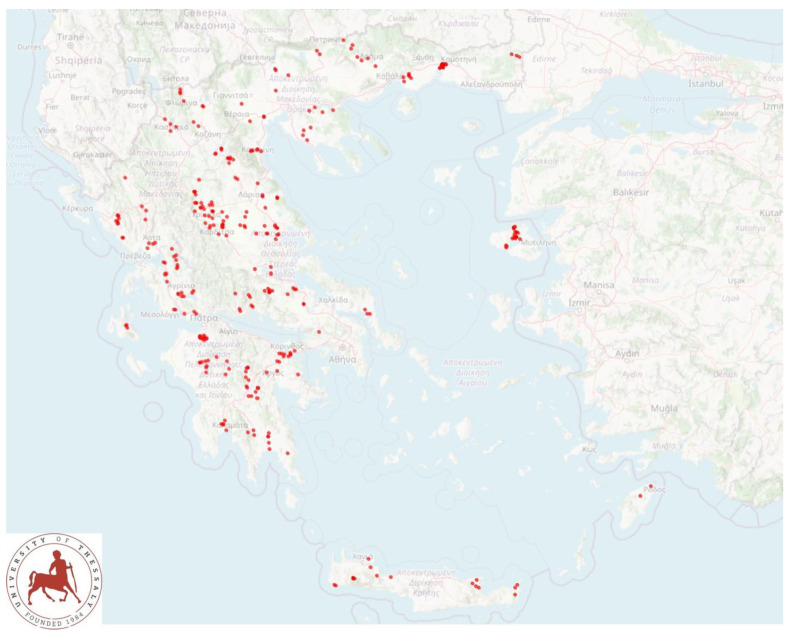
The location of 325 sheep flocks around Greece, which were visited for faecal sampling.

A cross-sectional study involving 325 flocks was performed from April 2019 to July 2020 and covered all the 13 administrative regions of Greece (Figure 1). Flocks were included in the study on a convenience basis. In brief, veterinarians active in small ruminant health management around Greece (*n* = 47), were contacted and asked to collaborate in the investigation. Flocks were selected by these veterinarians on a convenience basis (the willingness of farmers to accept a visit by university personnel for an interview and sample collection). The investigators (authors D.T.L., C.K.M. and G.C.F.) visited all the flocks for sample collection. Samples were collected directly from the rectum of adult ewes on the farms, as described in detail by Lianou et al. [15]. In each flock, 20, 30, 40 or 50 ewes (for flocks with ≤165, 166–330, 331–500 or >500 ewes, respectively) were selected for sampling. The management practices applied in the flocks were recorded during an interview with the shepherd by means of a detailed structured questionnaire [16].

### 2.2. Laboratory Examinations

The transportation of the samples to the laboratory was provided by the investigators, by car. Samples collected from farms in the islands were transported as accompanying luggage by airplane (Crete, Lesvos and Rhodes) or by boat (Cephalonia).

Parasitological examinations started within 48 h after the collection of samples. Initially, 5 g of each of the individual animal’s faecal sample from a farm was taken and mixed to form the pooled faecal sample from the farm, which was then processed in a homogenizing blender. The usefulness of pooling ovine faecal samples as a rapid procedure for the identification of gastrointestinal helminths at a farm level has been confirmed by Rinaldi et al. [17].

On the material from the pooled faecal samples, the following parasitological tests were performed: the McMaster technique (3 g), the flotation method (1 g), the sedimentation technique (1 g) and coproculture (remaining quantity). Each of the first three techniques were applied in quadruplicate samples (each 5 g) obtained from the pooled faecal sample, whilst coproculture was performed once.

The detection of a specific helminth at least once in the four times that each technique was performed, was considered to indicate the presence of the respective organism on the farm, and the farm was declared as ‘positive’ for that particular helminth.

In a proportion of the farms studied (91 flocks, 28.0% of all flocks in the study), the above examinations were also performed on the faecal samples from all individual animals sampled on those farms (i.e., additionally to the pooled faecal sample of the farm) (Tables S1 and S2).

### 2.3. Data Management and Analysis

Data were entered into Microsoft Excel and analysed using SPSS v. 21 (IBM Analytics, Armonk, NY, USA). A basic descriptive analysis was performed, and exact binomial confidence intervals (CIs) were obtained.

Farms were assigned into two cohorts: those in which anthelmintics had or had not been administered during the two months prior to sampling (46 (14.2%) and 279 (85.8%), respectively). Moreover, in order to evaluate potential associations with the location of farms, the 13 administrative regions of Greece were clustered into four main areas: North, Central, South and Islands, as detailed in Table S3 and presented in Figure S1.

In total, 27 variables (related to the infrastructure, animals, management and human resources in the flocks) were evaluated for potential association with the recovery of helminths from the pooled faecal samples (Table S4); these were either taken directly from the answers of the interview performed at the start of the visit or were calculated based on these answers. For each of these variables, categories were created according to the answers of the farmers.

In flocks, in which anthelmintics had not been administered during the two months prior to sampling (*n* = 279), the following six outcomes were considered: ‘presence of *Dicrocoelium dendriticum* in faecal samples’, ‘high (>300) epg counts in faecal samples’, ‘high proportion (>63%) of *Teladorsagia* spp. Among Trichostrongylidae helminths in faecal samples’, ‘high proportion (>29%) of *Haemonchus contortus* among Trichostrongylidae helminths in faecal samples’, ‘presence of *Trichuris* spp. in faecal samples’ and ‘presence of lungworms in faecal samples’. For epg counts, the value of 300 epg was defined, because in previous studies it had been considered as the threshold above which anthelmintic treatments should be performed [18] and was also found to be associated with the increased prevalence of subclinical mastitis [7] in dairy sheep in Greece. For the proportion of *Teladorsagia* spp., the threshold (63%) was defined, because in a recent study it emerged as a predictor affecting the composition of milk in dairy sheep [5]. Finally, for the proportion of *H. contortus*, the threshold (29%) was defined as the average proportion of the helminth in samples from all the untreated flocks in the present study.

Exact binomial CIs were obtained. Initially, a standard univariable analysis was performed (Pearson’s chi-squared test and a simple logistic regression). Subsequently, multivariable models were created (mixed-effects logistic regressions with flocks as the random effect), with all variables that achieved a significance of *p* ≤ 0.2 in the univariable analysis (Table S5). Backwards elimination was then applied, and variables were progressively removed. The *p* value of removal of a variable was assessed by the likelihood ratio test, and for those with a *p* value of >0.2, the variable with the largest probability was removed. This process was repeated until no variable could be removed with a *p* value of >0.2. The variables required for the final multivariable tests for each of the six outcomes are presented in Table S5. Statistical significance was set at *p* < 0.05.

## 3. Results

### 3.1. Descriptive Results

Parasitic elements were found in faecal samples from 302 flocks (92.9%; 95% confidence intervals (CIs): 89.6–95.2%). Specifically, and based on the faecal parasitological methods performed, the following helminths were detected in samples from at least one flock: *D. dendriticum* (16.7% of flocks), *Fasciola hepatica* (0.6% of flocks), *Paramphistomum cervi* (2.2%), *Moniezia* spp. (18.8%), Trichostrongylidae (85.5%) (*Teladorsagia* spp., *H. contortus*, *Trichostrongylus* spp., *Chabertia* spp., *Cooperia* spp. and *Bunostomum* spp.), *Nematodirus* spp. (18.8%), *Strongyloides papillosus* (7.1%), *Trichuris* spp. (20.0%) and lungworms (17.8%) (Table 1). The mean Trichostrongylidae counts across all flocks in the study were 215 ± 13 epg; in samples from 66 flocks (20.3%; 95% CIs: 16.3–25.0%), counts were >300 epg.

There were differences between flocks that had or had not received anthelmintics during the two months prior to sampling, which referred to the prevalence and severity of infections with gastrointestinal nematode helminths (Table 2). Mean Trichostrongylidae counts in the respective flocks were 243 ± 14 epg versus 49 ± 11 epg (*p* < 0.0001). The 66 flocks with counts >300 epg had not received anthelmintics (24.5% of untreated flocks; *p* < 0.0001) (Table 2).

There were also differences between flocks, depending on their geographical location in the country. In general, infections were found to be more severe in the northern part of the country, where epg counts were higher and recoveries of Trichostrongylidae (*Teladorsagia* spp. and *H. contortus*), as well as of *Nematodirus* spp., were more frequent (Table 3). Significant differences between geographical areas were evident among flocks that had or had not received anthelmintics; for example, the differences in epg counts between flocks in the four geographical areas were significant among both cohorts (*p* = 0.0005 for untreated and *p* = 0.013 for treated flocks) (Table S6).

### 3.2. Variables Associated with Presence of Helminths in Faecal Samples

The detailed results of the univariable analyses for the six outcomes considered are in Tables S7–S12.

**Figure 2:**
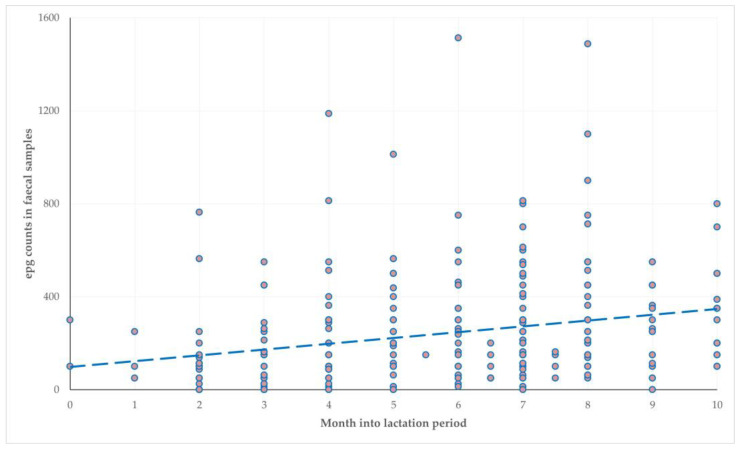
The association of the month into lactation period at sampling with epg counts in faecal samples from 279 sheep flocks in a countrywide investigation in Greece.

**Figure 3:**
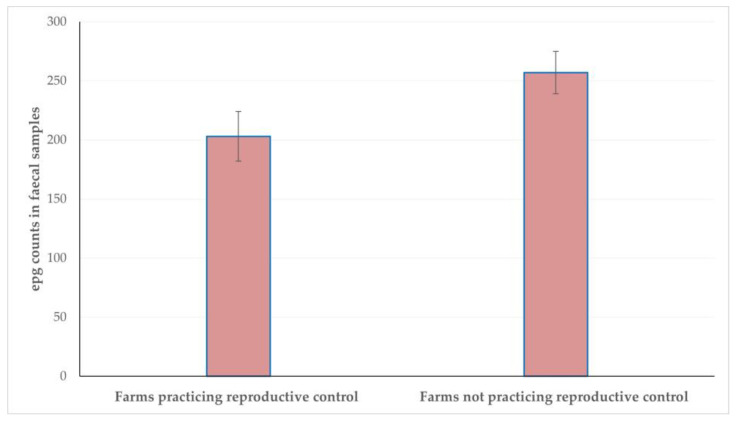
The association of the application of reproductive control practices with epg counts in faecal samples from 279 sheep flocks in a countrywide investigation in Greece.

During the multivariable analysis, for the outcome ‘high (>300) epg counts in faecal samples’, the following emerged as significant factors: (a) month into the lactation period at sampling (*p* = 0.0003) (Figure 2) and (b) application of reproductive control practices on the farm (*p* = 0.048) (Figure 3) (Table 4).

During the multivariable analysis, for the outcome ‘high proportion (>63%) of *Teladorsagia* spp. among Trichostrongylidae helminths in faecal samples’, the availability of straw bedding (*p* = 0.010) emerged as a significant factor (Table 5).

During the multivariable analysis, for the outcome ‘high proportion (>29%) of *H. contortus* among Trichostrongylidae helminths in faecal samples’, the age of the farmer (*p* = 0.040) emerged as a significant factor (Table 6).

During the multivariable analysis, for the outcome ‘presence of *Trichuris* spp. in faecal samples’, the provision of finished feed (concentrate) to animals throughout the year (*p* = 0.024) emerged as a significant factor (Table 7).

Finally, during the multivariable analysis, no significant factors emerged for the outcomes ‘presence of *D. dendriticum* in faecal samples’ (*p* > 0.09) and ‘presence of lungworms in faecal samples’ (*p* > 0.12).

## 4. Discussion

The findings provide, for the first time, a detailed presentation of the helminth infections in sheep flocks in Greece. 

Conventional parasitological techniques for helminth detection have been employed in this study and one may consider this as a potential limitation due to a possible underestimation of the presence of helminths in comparison to the use of molecular techniques. Nevertheless, it should be noted that such techniques are well-established and are used for helminth detection in clinical settings, which thus makes the present results of reference value for clinicians. Furthermore, for the trichostrongylid helminths, the results are provided at family level (Trichostrongylidae) and at genus level (*Teladorsagia* spp. (in Greece, the predominant species in sheep being *T. circumcincta*), *H. contortus*, *Trichostrongylus* spp., *Chabertia* spp., *Cooperia* spp. and *Bunostomum* spp.), as found by means of corpoculturing, used as the standard relevant method. The cumulative results for the Trichostrongylidae family helminths also relate to the results of epg counting, which is an important tool in parasitological examinations, as it confirms the severity of helminth infections. For respiratory helminths, for which results at genus or species level have not been provided, it is noted that the therapeutic and preventive approaches against all the lungworms infecting sheep are similar, independently of the species involved. In Greece, in previous studies, the following species have been identified in faecal samples from sheep: *Dictyocaulus filaria*, *Cystocaulus ocreatus*, *Protostrongylus rufescens*, *Muellerius capillaris* and *Neostrongylus linearis*.

First, the results revealed differences in helminth infections between areas of the country. Climatic differences between the geographical areas may be responsible for these. For example, the significantly higher prevalence of *Nematodirus* spp. can be related to the lower temperatures prevailing in locations with sheep flocks in the northern part of the country [19], given that the hatching of eggs from the helminth is facilitated by cold weather [20]. Moreover, the milder temperatures in locations with sheep flocks on the islands may have contributed to the high proportion of *H. contortus* among the Trichostrongylidae helminths found in those flocks. Management practices may have further contributed. The first cases of anthelmintic resistance in gastrointestinal nematodes in Greece were diagnosed in sheep on the islands [14]; since then, programs to prevent further development and spread of anthelmintic resistance have been intensively applied in the islands. Possibly, therefore, one may suggest that the success of these efforts was reflected in the low epg counts found in the flocks on the islands. In addition, the possibility of increased helminth infections in the northern part may reflect dissemination of parasitic forms by wildlife, which cross the borders of the country [21]. Cross-infections with various helminths between small ruminants and herbivore wildlife should also be considered as a consequence of the movements of animals, when they share the same habitats [22].

Second, a noteworthy finding was the detection of helminth infections in flocks despite the administration of anthelmintic treatment. Although the frequency and severity of trichostrongylid infections were significantly lower among treated flocks, there were still farms from which helminths were detected during the subsequent sampling. This suggests the presence of anthelmintic resistance in these flocks. However, the possibility of the inappropriate application of anthelmintic drugs cannot be ruled out, e.g., the administration at lower doses, which would result in reduced efficacy, as well as increasing the risk of the development of resistance. The findings suggest that anthelmintic resistance in this country seems to occur in various helminth species.

It is also noteworthy that for some helminths, there are problems with dosage regimes. For example, for *D. dendriticum*, which is the prevalent trematode in this country, the effective dose of albendazole is 20 mg per kg of bodyweight [23], but there are no data regarding withdrawal periods for sheep meat and milk after administration of that dose rate. Therefore, this dose rate would be infrequently applied in clinical practice and the flocks would remain untreated against this helminth; this was reflected in the lack of significant differences in the frequency of infection between flocks that had or had not received anthelmintic treatments. As liver trematode infections may predispose to pregnancy toxaemia [24], our findings highlight the need for the specific study of residues and the establishment of withdrawal periods for sheep milk and meat.

Third, the identification of predictors can provide a guide for applying the appropriate health management for controlling helminth infections. The frequency of high epg counts increased as the lactation period advanced; during the first 10–15 days after lambing, ewes may remain housed (thus provided with concentrate feed), for monitoring for post-parturient disorders and for supporting the establishment of the dam–newborn bond [25]. After that period, they would be grazing, which increases the risk of infection by trichostrongulid larvae. Moreover, at the second month post-lambing, milking starts, with the peak milk yield being achieved at the 4^th^ to 6^th^ month of the lactation period, which increases the stress of animals and thus the excretion of helminth eggs. The application of reproductive control has been associated with a generally better management of flocks [26]; in this way, the start of the lambing season can be programmed and the application of anthelmintic treatments can be finetuned to minimise helminth burdens at lambing; therefore, avoiding the peri-parturient egg-rise and the consequent high helminth burdens.

Other factors, e.g., the availability of straw bedding and the continuous provision of concentrate feed for the animals, would be related to decreasing the risk of infection by parasites. The availability of straw bedding can reduce floor humidity within animal barns, which would contribute to the reduced larval viability of *Teladorsagia* spp. [27], thus reducing the risk of infection in animals. The provision of concentrate feed throughout the year can be associated with a smaller risk of infection by *Trichuris* spp., as the consequent shorter grazing time and increased protein supplementation contribute to reducing nematode establishment in the gastrointestinal tract.

For infection by *H. contortus*, the age of the farmers was identified as a predictor. The productivity of farms has been reported to decrease as farmers get older [28,29]. This could be associated with the findings of a study performed in New Zealand, which indicated that shepherds aged over 50 years used fewer health management tools (e.g., vaccinations or anthelmintic treatments) than younger farmers [30]. This could impede the control of diseases needing complex management, e.g., helminth infections. One may hypothesise that older farmers would be using older-generation anthelmintics, e.g., benzimidazoles, for which extensive resistance has been found in *H. contortus* in Greece [31]. There may be also a further association of the farmers’ age with the recovery of *H. contortus*: older farmers would usually administer benzimidazoles in bolus form, the traditional form of antiparasitics, which nevertheless are manufactured for standard dosing to 40 kg-animals [32], whilst contemporary animals in the flocks in Greece, usually weigh over 50 to 60 kg [33]. This may lead to a systematic underdosing against trichostrongylids, potentially causing the widespread resistance of *H. contortus* in this country [31].

## 5. Conclusions

This study presents the extent of helminth infections in sheep flocks in Greece. The results indicate some further facets of the problem of anthelmintic resistance in the country. The management factors that have emerged (as detailed according to the respective infections) should be considered as important for the control of the respective infections, by reducing the presence of helminth infections, therefore, further controlling the development of anthelmintic resistance. Moreover, on farms where these factors are applied as standard practices, they can be included as specific preventive measures against the respective infections.

## Figures and Tables

**Table 1:** The cumulative results of parasitological examinations in pooled faecal samples from 325 sheep flocks in a countrywide investigation in Greece.

	Frequency (Proportion) of Flocks in Which the Presence of Parasitic Elements of the Following Helminths Was Detected															
	Dd ^1^	Fh	Pc	Mon	Trich fam	Tel	Hc	Trich spp.	Chab	Coop	Bun	Nem	Sp	Trichur	Lung	epg ^2^ > 300
All flocks (*n* = 325)	54 (16.7%)	2 (0.6%)	7 (2.2%)	61 (18.8%)	278 (85.5%)	278 (85.5%)	277 (85.2%)	257 (79.1%)	206 (63.4%)	140 (43.1%)	65 (20.0%)	61 (18.8%)	23 (7.1%)	65 (20.0%)	58 (17.8%)	66 (20.3%)
	**Mean ± Standard Error of the Mean Parameter in Faecal Samples**															
	epg		Proportion (%) Tela		Proportion (%) Haem		Proportion (%) Tricho			Proportion (%) Chab		Proportion (%) Coop		Proportion (%) Buno		
All flocks (*n* = 325)	214 ± 13		54.3 ± 1.3		27.4 ± 0.7		2.3 ± 0.1			0.8 ± 0.1		0.5 ± 0.1		0.2 ± 0.1		

^1^ Dd = *D. dendriticum*, Fh = *F. hepatica*, Pc = *Paramphistomum cervi*, Mon = *Moniezia* spp., Trich fam = Trichostrongylidae, Tel = *Teladorsagia* spp., Hc = *H. contortus*, Trich spp. = *Trichostrongylus* spp., Chab = *Chabertia* spp., Coop = *Cooperia* spp., Bun = *Bunostomum* spp., Nem = *Nematodirus* spp., Sp = *St. papillosus*, Trichur = *Trichuris* spp., Lung = lungworms. ^2^ Trichostrongulidae eggs per gram.

**Table 2:** The results of parasitological examinations in pooled faecal samples from 325 sheep flocks in a countrywide investigation in Greece, in accord with the administration of anthelmintic treatment during the two months prior to sampling.

	Frequency (Proportion) of Flocks in Which the Presence of Parasitic Elements of the Following Helminths Was Detected															
	Dd ^1^	Fh	Pc	Mon	Trich fam	Tel	Hc	Trich spp.	Chab	Coop	Bun	Nem	Sp	Trichur	Lung	epg ^2^ > 300
Untrea -ted flocks ^3^ (*n* = 279)	49 (17.6%)	1 (0.4%)	5 (1.8%)	53 (19.0%)	261 (93.5%)	261 (93.5%)	260 (93.2%)	243 (87.1%)	194 (69.5%)	128 (45.9%)	64 (22.9%)	55 (19.7%)	19 (6.8%)	61 (21.8%)	54 (19.4%)	66 (24.5%)
Treated flocks ^3^ (*n* = 46)	5 (10.9%)	1 (0.2%)	2 (4.3%)	8 (17.4%)	17 (37.0%)	17 (37.0%)	17 (37.0%)	14 (30.4%)	12 (26.1%)	9 (19.6%)	1 (2.2%)	6 (13.0%)	4 (8.7%)	4 (8.7%)	4 (8.7%)	0 (0.0%)
*p*-value ^4^	0.26	0.14	0.27	0.80	<0.0001	<0.001	<0.001	0.001	0.008	0.037	0.43	0.28	0.64	0.038	0.08	<0.0001
	**Mean ± Standard Error of the Mean Parameter in Faecal Samples**															
	epg		Proportion (%) Tela		Proportion (%) Haem		Proportion (%) Tricho			Proportion (%) Chab		Proportion (%) Coop		Proportion (%) Buno		
Untrea-ted flocks (*n* = 279)	242 ± 13		59.5 ± 1.1		29.8 ± 0.7		2.6 ± 0.1			0.9 ± 0.0		0.6 ± 0.0		0.2 ± 0.0		
Treated flocks (*n* = 46)	43 ± 11		22.4 ± 4.4		13.1 ± 2.6		0.8 ± 0.2			0.3 ± 0.1		0.3 ± 0.1		0.2 ± 0.0		
*p*-value ^4^	<0.0001		<0.0001		<0.0001		<0.0001			<0.0001		0.004		0.001		

^1^ Dd = *D. dendriticum*, Fh = *F. hepatica*, Pc = *Paramphistomum cervi*, Mon = *Moniezia* spp., Trich fam = Trichostrongylidae, Tel = *Teladorsagia* spp., Hc = *H. contortus*, Trich spp. = *Trichostrongylus* spp., Chab = *Chabertia* spp., Coop = *Cooperia* spp., Bun = *Bunostomum* spp., Nem = *Nematodirus* spp., Sp = *St. papillosus*, Trichur = *Trichuris* spp., Lung = lungworms. ^2^ Trichostrongulidae eggs per gram. ^3^ Untreated/Treated flocks = Flocks that had not/had received anthelmintics during the two months prior to sampling. ^4^ *p*-value = for comparison between untreated and treated flocks.

**Table 3:** The results of parasitological examinations in pooled faecal samples from 325 sheep flocks in a countrywide investigation in Greece, in accord with the geographical area of the country.

	Frequency (Proportion) of Flocks in Which the Presence of Parasitic Elements of the Following Helminths Was Detected															
	Dd ^1^	Fh	Pc	Mon	Trich fam	Tel	Hc	Trich spp.	Chab	Coop	Bun	Nem	Sp	Trichur	Lung	epg ^2^ > 300
North ^3^ (*n* = 87)	12 (13.8%)	0 (0.0%)	0 (0.0%)	14 (16.1%)	87 (100.0%)	87 (100.0%)	87 (100.0%)	82 (94.3%)	64 (73.6%)	46 (52.9%)	22 (25.3%)	28 (32.2%)	6 (6.9%)	19 (21.8%)	21 (24.1%)	32 (36.8%)
Central (*n* = 127)	24 (18.9%)	1 (0.8%)	2 (1.6%)	27 (21.2%)	104 (81.9%)	104 (81.9%)	103 (81.1%)	95 (74.8%)	77 (60.6%)	52 (40.9%)	24 (18.8%)	22 (17.3%)	11 (8.7%)	24 (18.9%)	20 (15.7%)	15 (11.8%)
South (*n* = 68)	9 (13.2%)	1 (1.5%)	4 (5.9%)	13 (19.1%)	45 (66.2%)	45 (66.2%)	45 (66.2%)	42 (61.8%)	35 (51.5%)	21 (30.9%)	8 (11.8%)	10 (14.7%)	5 (7.4%)	14 (20.6%)	9 (13.2%)	16 (23.5%)
Islands (*n* = 43)	9 (20.9%)	0 (0.0%)	1 (2.3%)	7 (16.3%)	42 (97.7%)	42 (97.7%)	42 (97.7%)	38 (88.4%)	30 (69.8%)	18 (41.9%)	11 (25.6%)	1 (2.3%)	1 (2.3%)	8 (18.6%)	8 (18.6%)	3 (7.0%)
*p*-value ^4^	0.55	0.64	0.09	0.78	<0.0001	<0.0001	<0.0001	0.001	0.0005	0.08	0.63	0.0003	0.58	0.95	0.29	<0.0001
	**Mean ± Standard Error of the Mean Parameter in Faecal Samples**															
	epg		Proportion (%) Tela		Proportion (%) Haem		Proportion (%) Tricho			Proportion (%) Chab		Proportion (%) Coop		Proportion (%) Buno		
North (*n* = 87)	296 ± 23		63.0 ± 0.9		32.1 ± 0.9		3.0 ± 0.2			1.0 ± 0.8		0.7 ± 0.1		0.3 ± 0.1		
Central (*n* = 127)	164 ± 16		51.7 ± 2.3		26.6 ± 1.3		2.1 ± 0.2			0.8 ± 0.1		0.5 ± 0.1		0.2 ± 0.0		
South (*n* = 68)	224 ± 42		43.0 ± 3.9		20.3 ± 2.0		1.7 ± 0.3			0.7 ± 0.1		0.4 ± 0.1		0.1 ± 0.0		
Islands (*n* = 43)	177 ± 18		61.8 ± 1.9		31.5 ± 1.4		2.6 ± 0.4			0.9 ± 0.1		0.6 ± 0.1		0.3 ± 0.1		
*p*-value ^4^	0.0004		<0.0001		<0.0001		0.001			0.035		0.34		0.63		

^1^ Dd = *D. dendriticum*, Fh = *F. hepatica*, Pc = *Paramphistomum cervi*, Mon = *Moniezia* spp., Trich fam = Trichostrongylidae, Tel = *Teladorsagia* spp., Hc = *H. contortus*, Trich spp. = *Trichostrongylus* spp., Chab = *Chabertia* spp., Coop = *Cooperia* spp., Bun = *Bunostomum* spp., Nem = *Nematodirus* spp., Str = *St. papillosus*, Trichur = *Trichuris* spp., Lung = lungworms. ^2^ Trichostrongulidae eggs per gram. ^3^ Details of the geographical areas in Table S3 and Figure S1. ^4^ *p*-value = for comparison between flocks in the four geographical areas.

**Table 4:** The results of the multivariable analysis for high (>300) epg counts in faecal samples from 279 sheep flocks in a countrywide investigation in Greece.

Variables	Odds Ratio ^1^ (95% Confidence Intervals)	*p* Value
Month into the lactation period at sampling		0.0003
0–1 (0/5: 0.0%)	reference	-
2–5 (19/117: 16.2%)	2.178 (0.116–41.011)	0.60
6–9 (39/141: 27.7%)	4.239 (0.229–78.463)	0.33
10 and thereafter (8/16: 50.0%)	11.000 (0.522–231.625)	0.12
Application of reproductive control practices on the farm		0.048
Yes (13/77 = 16.9%)	reference	-
No (53/202 = 26.2%)	1.751 (0.893–3.435)	0.10

^1^ odds ratio calculated against the lowest prevalence associations of the variable.

**Table 5:** The results of the multivariable analysis for a high proportion (>63%) of *Teladorsagia* spp. among Trichostrongylidae helminths in faecal samples from 279 sheep flocks in a countrywide investigation in Greece.

Variables	Odds Ratio ^1^ (95% Confidence Intervals)	*p* Value
Availability of straw bedding		0.010
Yes (70/227 = 30.8%)	reference	-
No (24/52 = 46.8%)	1.922 (1.041–3.551)	0.037

^1^ odds ratio calculated against the lowest prevalence associations of the variable.

**Table 6:** The results of the multivariable analysis for a high proportion (>29%) of *H. contortus* among Trichostrongylidae helminths in faecal samples from 279 sheep flocks in a countrywide investigation in Greece.

Variables	Odds Ratio ^1^ (95% Confidence Intervals)	*p* Value
Age of farmer		0.040
≤50 years (88/166 = 53.0%)	reference	-
>50 years (72/113 = 63.7%)	1.557 (0.954–2.540)	0.08

^1^ odds ratio calculated against the lowest prevalence associations of the variable.

**Table 7:** The results of the multivariable analysis for the presence of *Trichuris* spp. in faecal samples from 279 sheep flocks in a countrywide investigation in Greece.

Variables	Odds Ratio ^1^ (95% Confidence Intervals)	*p* Value
Provision of finished feed (concentrate) to animals throughout the year		0.024
Yes (54/260 = 20.8%)	reference	-
No (7/19 = 36.8%)	2.225 (0.836–5.924)	0.11

^1^ odds ratio calculated against the lowest prevalence associations of the variable.

## Data Availability

Most data presented in this study are in the Supplementary Materials. The remaining data are available upon request from the corresponding author. The data are not publicly available, as they form part of the PhD thesis of the first author, which has not yet been examined, approved, and uploaded in the official depository of Ph.D. theses from Greek universities.

## Supplementary Materials

The following supporting information can be downloaded at: https://www.mdpi.com/article/10.3390/microorganisms11030571/s1, Table S1: The presence of helminths in faecal samples from individual animals in sheep flocks in Greece, in which such parasites had been previously found in pooled faecal samples; Table S2: The presence of helminths in faecal samples from individual animals in sheep flocks in Greece, in which such parasites had not been previously found in pooled faecal samples; Table S3: The geographical areas of Greece in which administrative regions and regional units of the country were clustered, for characterizing the location of 325 sheep flocks from which faecal samples were collected; Table S4: The variables evaluated for potential association with the presence of helminths in faecal samples from 279 sheep flocks in Greece; Table S5: Details of the multivariable models employed for the evaluation of the presence of helminths in faecal samples from 279 sheep flocks in Greece; Table S6: The results of parasitological examinations in pooled faecal samples from 325 sheep flocks in a countrywide investigation in Greece, in accord with the geographical area of the country and the administration of anthelmintic treatment during the two months prior to sampling; Table S7: The associations of the presence of *Dicrocoelum dendriticum* in faecal samples from 279 sheep flocks in Greece, as found in the univariable analysis; Table S8: The associations of high (>300) epg counts in faecal samples from 279 sheep flocks in Greece, as found in the univariable analysis; Table S9: The associations of a high proportion (>63%) of *Teladorsagia* spp. among Trichostrongylidae helminths in faecal samples from 279 sheep flocks in Greece, as found in the univariable analysis; Table S10: The associations of a high proportion (>29%) of *Haemonchus contortus* among Trichostrongylidae helminths in faecal samples from 279 sheep flocks in Greece, as found in the univariable analysis; Table S11: The associations of the presence of *Trichuris* spp. in faecal samples from 279 sheep flocks in Greece, as found in the univariable analysis; Table S12: The associations of the presence of lungworms in faecal samples from 279 sheep flocks in Greece, as found in the univariable analysis; Figure S1: A map of Greece indicating the geographical areas of the country, in which the administrative regions and regional units of the country were clustered, for characterizing the location of 325 sheep flocks from which faecal samples were collected.
