# Supplementary material for: Helminth Infections in Dairy Sheep Found in an Extensive Countrywide Study in Greece and Potential Predictors for Their Presence in Faecal Samples
Source: Microorganisms. 2023 Feb 24;11(3):571. doi: 10.3390/microorganisms11030571 (PMC10055712; doi:10.3390/microorganisms11030571)
Supplement: Supplementary file 1 [file microorganisms-11-00571-s001.zip › microorganisms-2233604-supplementary.pdf]

Helminth Infections in Dairy Sheep Found in an Extensive Countrywide Study in Greece and Potential Predictors for Their Presence in Faecal Samples

Daphne T. Lianou, Konstantinos Arsenopoulos, Charalambia K. Michael, Vasia S. Mavrogianni, Elias Papadopoulos, and George C. Fthenakis

Table S1. Presence of helminths in faecal samples from individual animals in sheep flocks, in which such parasites had been found in pooled faecal samples, in Greece.

Helminths	No. of farms in which helminths were found in pooled faecal samples and in which faecal samples from individual animals were also examined	No. of animals present in these farms	No. of animals sampled in these farms	No. of animals in the faecal samples of which helminths were found	Overall prevalence of recovery of helminths in samples from individual animals	Among farms median prevalence of recovery of helminths in samples from individual animals
Dicrocoelium dendriticum	13 (24.1% [1])	2,957	350	150	42.9%	40.0%
Fasciola hepatica	0 (0.0%)	0	0	0	0.0%	n/a
Paramphistomum cervi	2 (28.6%)	410	50	7	14.0%	21.8%
Moniezia spp.	20 (32.8%)	5,809	560	265	47.3%	48.3%
Trichostrongylidae	77 (27.7%)	19,842	2,210	1,813	82.0%	93.5%
Nematodirus spp.	13 (21.3%)	4,573	390	183	46.9%	45.0%
Stronguloides papillosus	8 (34.8%)	3,789	250	79	31.6%	33.8%
Trichuris spp.	20 (30.8%)	6,027	570	173	30.4%	30.0%
Lungworms	18 (31.0%)	3,859	470	230	48.9%	49.0%

[1] figures in brackets indicate the proportion of these flocks among all those from which respective helminths had been found in pooled faecal samples.

Table S2. Presence of helminths in faecal samples from individual animals in sheep flocks, in which such parasites had not been found in pooled faecal samples, in Greece.

Helminths	No. of farms in which helminths were not found in pooled faecal samples and in which faecal samples from individual animals were also examined	No. of animals present in these farms	No. of animals sampled in these farms	No. of animals in the faecal samples of which helminths were found	Overall prevalence of recovery of helminths in samples from individual animals	Among farms median prevalence of recovery of helminths in samples from individual animals
Dicrocoelium dendriticum	78 (28.8% [1])	21,210	2,250	0	0.0%	0.0%
Fasciola hepatica	0 (0.0%)	24,167	2,600	0	0.0%	0.0%
Paramphistomum cervi	89 (28.0%)	23,757	2,550	0	0.0%	0.0%
Moniezia spp.	71 (26.9%)	18,358	2,040	0	0.0%	0.0%
Trichostrongylidae	14 (29.8%)	4,325	390	0	0.0%	0.0%
Nematodirus spp.	78 (29.5%)	19,594	2,210	0	0.0%	0.0%
Stronguloides papillosus	83 (27.5%)	20,378	2,350	0	0.0%	0.0%
Trichuris spp.	71 (27.3%)	18,140	2,030	0	0.0%	0.0%
Lungworms	73 (29.2%)	20,308	2,130	0	0.0%	0.0%

[1] figures in brackets indicate the proportion of these flocks among all those from which respective helminths had not been found in pooled faecal samples.

Table S3. Geographical areas of Greece (n = 4), in which administrative regions and regional units of the country were clustered, for characterizing location of 325 sheep flocks from which faecal samples were collected.

**Central**
Region of Central Greece
Region of Thessaly
From the region of Epirus: regional unit of Arta and regional unit of Preveza
From the region of Western Greece: regional unit of Aetolia-Acarnania
**Islands**
Region of Crete
Region of Ionian islands
Region of North Aegean
Region of South Aegean
**North**
Region of Central Macedonia
Region of Eastern Maecedonia and Thrace
Region of Western Macedonia
From the region of Epirus: regional unit of Ioannina and regional unit of Thesprotia
**South**
Region of Attica
Region of Peloponnese
Region of Western Macedonia
From the region of Western Greece: regional unit of Achaea and regional unit of Elis

Figure S1. Map of Greece indicating the geographic areas of the country (n = 4), in which administrative regions and regional units of the country were clustered, for characterizing location of 325 sheep flocks from which faecal samples were collected (blue: Central, green: Islands, yellow: North, red: South).

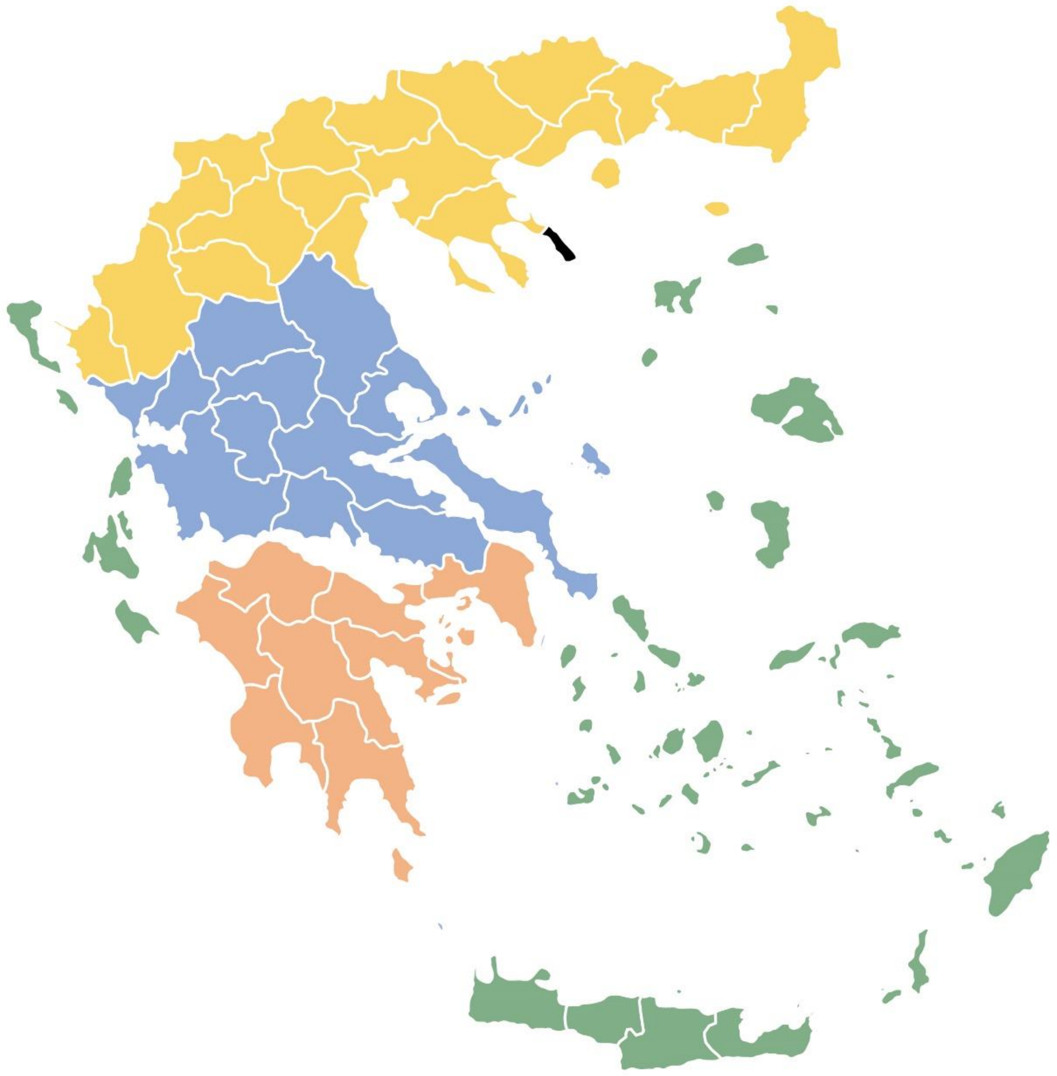

Table S4. Variables (n = 27) evaluated for potential association with presence of helminths in faecal samples from 279 sheep flocks in Greece.

Management system applied in the farm (description according to EFSA classification)
Years at the location (no. of years)
Month into the lactation period at sampling (month)
Availability of a main building for animals (yes / no)
Material of the floor of the barn (soil / other)
Availability of straw bedding (yes / no)
Annual frequency of removal / clean-up of the straw bedding (no. of occasions)
Grazing practiced (yes / no)
Grazing land available to animals (acres per animal)
No. of female animals in the farm (no.)
Breed of ewes (description)
Month of the start of the lambing season (description)
Collaboration with a veterinarian (yes / no)
Use of laboratory diagnostic examinations in faecal samples preventively (yes / no)
Total visits made annually by veterinarians to the farm during the preceding season (no.)
Application of reproductive control practices in the farm (yes / no)
Nutritional modifications before the lambing period (yes / no)
Age of lamb removal from their dams (days)
Seasonal transfer of animals to other site (yes / no)
Annual frequency of systemic disinfections in the farm (no.)
Shearing of animals (yes / no)
Provision of finished feed (concentrate) to animals throughout the year (yes / no)
Age of the farmer (years)
Length of animal farming experience of the farmer (years)
Education of the farmer (description)
Farmer by profession (yes / no)
Presence of working staff in the flock (yes / no)

Table S5. Details of multivariable models employed for the evaluation of the presence of helminths in faecal samples from 279 sheep flocks in Greece.

Outcome	Variables offered to the multivariable models (*n*)	Variables required in the final models
Presence of *Dicrocoelium dendriticum* in faecal samples	4	(a) Availability of a main building for animals, (b) Material of the floor of the barn, (c) Seasonal transfer of animals to other site
High (> 300) epg counts in faecal samples	7	(a) Month into the lactation period at sampling, (b) No. of female animals in the farm, (c) Collaboration with a veterinarian, (d) Application of reproductive control practices in the farm, (e) Farmer by profession
High proportion (> 63%) of *Teladorsagi*a spp. among Trichostrongylidae helminths in faecal samples	7	(a) Availability of straw bedding, (b) Grazing practiced, (c) Breed of ewes, (d) Nutritional modifications before the lambing period, (e) Shearing of animals, (f) Age of the farmer
High proportion (> 29%) of *Haemonchus contortus* among Trichostrongylidae helminths in faecal samples'	9	(a) Month into the lactation period at sampling, (b) Availability of a main building for animals, (c) Availability of straw bedding, (d) Grazing land available to animals, (e) Breed of animals, (f) Seasonal transfer of animals to other site, (g) Age of the farmer
Presence of *Trichuris* spp. in faecal samples	7	(a) Month of the start of the lambing season, (b) Total visits made annually by veterinarians to the farm during the preceding season, (c) Provision of finished feed (concentrate) to animals throughout the year, (d) Age of the farmer
Presence of lungworms in faecal samples	4	(a) Availability of a main building for animals, (b) Shearing of animals, (c) Provision of finished feed (concentrate) to animals throughout the year, (d) Farmer by profession

Table S6. Results of parasitological examinations in pooled faecal samples from 325 sheep flocks in a countrywide investigation in Greece, in accord with the geographical area of the country and the administration of anthelmintic treatment during the two months prior to sampling.

Untreated flocks [1]

	Frequency (proportion) of flocks in which presence of parasitic elements of the following helminths was detected															
	Dd [2]	Fh	Pc	Mon	Trich fam	Tel	Hc	Trich spp.	Chab	Coop	Bun	Nem	Sp	Trichur	Lung	epg [3] > 300
North [4] (*n* = 84)	12 (14.3%)	0 (0.0%)	0 (0.0%)	13 (15.5%)	84 (100.0%)	84 (100.0%)	84 (100.0%)	79 (94.0%)	61 (72.6%)	43 (51.2%)	22 (26.2%)	28 (33.3%)	6 (7.1%)	19 (22.6%)	20 (23.8%)	32 (38.1%)
Central (*n* = 105)	22 (21.0%)	1 (1.0%)	2 (1.9%)	22 (21.0%)	98 (93.3%)	98 (93.3%)	98 (93.3%)	91 (86.7%)	74 (70.5%)	48 (45.7%)	24 (22.9%)	18 (17.1%)	9 (8.6%)	22 (21.0%)	18 (17.1%)	15 (14.3%)
South (*n* = 47)	6 (12.8%)	0 (0.0%)	2 (4.3%)	11 (23.4%)	37 (78.5%)	37 (78.5%)	37 (78.5%)	35 (74.4%)	29 (61.7%)	19 (40.4%)	7 (14.9%)	8 (17.0%)	3 (6.4%)	12 (25.5%)	8 (17.0%)	16 (34.0%)
Islands (*n* = 43)	9 (20.9%)	0 (0.0%)	1 (2.3%)	7 (16.3%)	42 (97.7%)	42 (97.7%)	42 (97.7%)	38 (88.3%)	30 (69.8%)	18 (41.9%)	11 (25.6%)	1 (2.3%)	1 (2.3%)	8 (18.6%)	8 (18.6%)	3 (7.0%)
p-value [5]	0.47	0.65	0.36	0.63	< 0.0001	< 0.0001	< 0.0001	0.016	0.62	0.62	0.49	0.0003	0.59	0.87	0.66	< 0.0001

	Mean ± standard error of the mean of parametre in faecal samples						
	epg	Proportion (%) Tela	Proportion (%) Haem	Proportion (%) Tricho	Proportion (%) Chab	Proportion (%) Coop	Proportion (%) Buno
North (*n* = 87)	301 ± 23	63.3 ± 0.9	31.9 ± 0.9	3.0 ± 0.2	1.0 ± 0.1	0.6 ± 0.1	0.1 ± 0.1
Central (*n* = 127)	190 ± 16	59.2 ± 1.6	30.0 ± 1.1	2.4 ± 0.2	0.9 ± 0.1	0.6 ± 0.1	0.2 ± 0.0
South (*n* = 68)	311 ± 47	51.2 ± 3.4	24.0 ± 1.8	2.1 ± 0.3	0.8 ± 0.1	0.5 ± 0.1	0.1 ± 0.1
Islands (*n* = 43)	177 ± 18	61.8 ± 1.9	31.6 ± 1.4	2.6 ± 0.4	0.9 ± 0.1	0.6 ± 0.1	0.3 ± 0.1
p-value	0.0007	0.02	0.001	0.13	0.67	0.81	0.83

Treated flocks

	Frequency (proportion) of flocks in which presence of parasitic elements of the following helminths was detected															
	Dicr	Fasc	Para	Moni	Tr/idae	Tela	Haem	Tricho	Chab	Coop	Buno	Nema	Stro	Trichu	Lung	epg[2] > 300
North (*n* = 3)	0 (0.0%)	0 (0.0%)	0 (0.0%)	1 (33.3%)	3 (100.0%)	3 (100.0%)	3 (100.0%)	3 (100.0%)	3 (100.0%)	3 (100.0%)	0 (0.0%)	0 (0.0%)	0 (0.0%)	0 (0.0%)	1 (33.3%)	0 (0.0%)
Central [3] (*n* = 22)	2 (9.1%)	0 (0.0%)	0 (0.0%)	5 (22.7%)	6 (27.3%)	6 (27.3%)	6 (27.3%)	4 (18.2%)	3 (13.6%)	4 (18.2%)	0 (0.0%)	4 (18.2%)	2 (9.1%)	2 (9.1%)	2 (9.1%)	0 (0.0%)
South (*n* = 21)	3 (14.3%)	1 (4.8%)	2 (9.5%)	2 (9.5%)	8 (38.1%)	8 (38.1%)	8 (38.1%)	7 (33.3%)	6 (28.6%)	2 (9.5%)	1 (4.8%)	2 (9.5%)	2 (9.5%)	2 (9.5%)	1 (4.8%)	0 (0.0%)

Islands (*n* = 0)	n/a	n/a	n/a	n/a	n/a	n/a	n/a	n/a	n/a	n/a	n/a	n/a	n/a	n/a	n/a	n/a
p-value	0.71	0.54	0.29	0.39	0.049	0.049	0.049	0.014	0.006	0.001	0.54	0.55	0.86	0.86	0.26	n/a

	Mean ± standard error of the mean of parametre in faecal samples						
	epg	Proportion (%) Tela	Proportion (%) Haem	Proportion (%) Tricho	Proportion (%) Chab	Proportion (%) Coop	Proportion (%) Buno
North (*n* = 3)	163 ± 15	54.7 ± 0.8	39.3 ± 0.6	3.3 ± 0.2	1.3 ± 0.1	1.3 ± 0.1	0.0 ± 0.0
Central (*n* = 22)	38 ± 6	16.0 ± 2.4	10.5 ± 1.6	0.5 ± 0.1	0.2 ± 0.1	0.2 ± 0.1	0.0 ± 0.0
South (*n* = 21)	30 ± 7	24.6 ± 3.9	12.1 ± 2.0	0.8 ± 0.2	0.3 ± 0.1	0.1 ± 0.1	0.1 ± 0.0
Islands (*n* = 0)	n/a	n/a	n/a	n/a	n/a	n/a	n/a
p-value	0.013	0.10	0.026	0.002	0.005	0.002	0.56

1 Untreated / Treated flocks = Flocks that had not / had received anthelmintics during the two months prior to sampling.
2 Dd = *Dicrocoelium dendriticum*, Fh = *Fasciola hepatica*, Pc = *Paramphistomum cervi*, Mon = *Moniezia* spp., Tric fam = Trichostrongylidae, Tel = *Teladorsagia* spp., Hc = *Haemonchus contortus*, Trich spp. = *Trichostrongylus* spp., Chab = *Chabertia* spp., Coop = *Cooperia* spp., Bun = *Bunostomum* spp., Nem = *Nematodirus* spp., Sp = *Strongyloides papillosus*, Trichur = *Trichuris* spp., Lung = lungworms.
3 Trichostrongulidae eggs per gram.
4 Details of the geographical areas in Table S3 and Figure S1.
5 *p*-value = for comparison between flocks in the four geographical areas.

Table S7. Associations of presence of *Dicrocoelium dendriticum* in faecal samples from 279 sheep flocks in Greece, as found in univariable analysis.

Number of farms								***p***
**Farms with presence of *Dicrocoelium dendriticum* in faecal samples (*n* = 49)**				**Farms with no presence of *Dicrocoelium dendriticum* in faecal samples (*n* = 230)**				
Management system applied in the farm								
Intensive	Semi-intensive	Semi-extensive	Extensive	Intensive	Semi-intensive	Semi-extensive	Extensive	
7	22	16	4	29	106	76	19	0.99
Years at the present location								
0 – 2	3 – 5	> 5		0 – 2	3 – 5	> 5		
2	5	42		3	25	202		0.41
Month into the lactation period at sampling								
0–1st	2nd–5th	6th–9th	After 9th	0–1st	2nd–5th	6th–9th	After 9th	
2	21	22	4	3	96	119	12	0.43
Availability of a main building for animals								
Yes	No			Yes	No			
46	3			226	4			0.07
Material of the floor of the barn								
Soil	Other			Soil	Other			
47	2			205	25			0.14
Availability of straw bedding								
Yes	No			Yes	No			
39	10			188	42			0.73
Annual frequency of removal / clean-up of the straw bedding								
1 – 2	> 2			1 – 2	> 2			
24	15			123	65			0.64
Grazing practiced								
Yes	No			Yes	No			
42	7			200	30			0.82
Grazing land available to animals								
0 – 0.5 acre per animal	> 0.5 acre per animal			0 – 0.5 acre per animal	> 0.5 acre per animal			
16	33			35	195			0.004
No. of female animals in the farm								
0 – 165	166 – 330	331 – 500	> 500	0 – 165	166 – 330	331 – 500	> 500	
10	16	13	10	65	80	47	38	0.57
Breed of ewes								
Cross-breeds	Greek breeds	Imported breeds		Cross-breeds	Greek breeds	Imported breeds		
8	22	19		31	100	99		0.81

Month of the start of the lambing season								
All year	Autumn	Winter	Spring-Summer	All year	Autumn	Winter	Spring-Summer	
1	37	11	0	14	173	38	5	0.38
Collaboration with a veterinarian								
Yes	No			Yes	No			
44	5			190	40			0.21
Use of laboratory diagnostic examinations in faecal samples preventively								
Yes	No			Yes	No			
2	47			20	210			0.28
Total visits made annually by veterinarians to the farm during the preceding season								
0 – 4	5 – 7	> 7		0 – 4	5 – 7	> 7		
22	13	14		101	59	70		0.97
Application of reproductive control practices in the farm								
Yes	No			Yes	No			
14	35			62	168			0.82
Nutritional modifications before the lambing period								
Yes	No			Yes	No			
31	18			162	68			0.32
Age of lamb removal from their dams								
≤ 40 days	41 – 60 days	> 60 days		≤ 40 days	41 – 60 days	> 60 days		
18	24	7		81	124	25		0.73
Seasonal transfer of animals to other site								
Yes	No			Yes	No			
13	36			34	196			0.046
Annual frequency of systemic disinfections in the farm								
0 – 9	> 9			0 – 9	> 9			
42	7			209	21			0.28
Shearing of animals								
Yes	No			Yes	No			
48	1			227	3			0.69
Provision of finished feed (concentrate) to animals throughout the year								
Yes	No			Yes	No			
46	3			214	16			0.83
Age of farmers								
Up to 50 years	Over 50 years			Up to 50 years	Over 50 years			
32	17			134	96			0.36

Length of previous animal farming experience of the farmer				
≤ 5 years	> 5 years	≤ 5 years	> 5 years	
7	42	49	181	0.27

Education of the farmer						
Primary education	Secondary or post-secondary education	Tertiary education	Primary education	Secondary or post-secondary education	Tertiary education	
6	39	4	40	156	34	0.25

Professional involvement in farming				
Full-time	Part-time	Full-time	Part-time	
45	4	204	26	0.52

Presence of working staff in the flock				
Yes	No	Yes	No	
16	33	86	144	0.53

Table S8. Associations of high (> 300) epg counts in faecal samples from 279 sheep flocks in Greece, as found in univariable analysis.

Number of farms								p
**Farms with high (> 300) epg counts in faecal samples (n = 66)**				**Farms with low (≤ 300) epg counts in faecal samples (n = 213)**				
Management system applied in the farm								
Intensive	Semi-intensive	Semi-extensive	Extensive	Intensive	Semi-intensive	Semi-extensive	Extensive	
10	27	23	6	26	101	69	17	0.81
Years at the present location								
0 – 2	3 – 5	> 5		0 – 2	3 – 5	> 5		
0	9	57		5	20	188		0.29
Month into the lactation period at sampling								
0–1st	2nd–5th	6th–9th	After 9th	0–1st	2nd–5th	6th–9th	After 9th	
0	19	39	8	5	98	102	8	0.006
Availability of a main building for animals								
Yes	No			Yes	No			
65	1			207	6			0.55
Material of the floor of the barn								
Soil	Other			Soil	Other			
57	9			195	18			0.21
Availability of straw bedding								
Yes	No			Yes	No			
50	16			177	36			0.18
Annual frequency of removal / clean-up of the straw bedding								
1 – 2	> 2			1 – 2	> 2			
30	20			117	60			0.43
Grazing practiced								
Yes	No			Yes	No			
55	11			187	26			0.35
Grazing land available to animals								
0 – 0.5 acre per animal	> 0.5 acre per animal			0 – 0.5 acre per animal	> 0.5 acre per animal			
22	44			76	137			0.73
No. of female animals in the farm								
0 – 165	166 – 330	331 – 500	> 500	0 – 165	166 – 330	331 – 500	> 500	
25	19	11	11	50	77	49	37	0.13
Breed of ewes								
Cross-breeds	Greek breeds	Imported breeds		Cross-breeds	Greek breeds	Imported breeds		
10	27	29		29	95	89		0.86

Month of the start of the lambing season								
All year	Autumn	Winter	Spring-Summer	All year	Autumn	Winter	Spring-Summer	
2	52	11	1	13	158	38	4	0.78
Collaboration with a veterinarian								
Yes	No			Yes	No			
59	7			175	38			0.16
Use of laboratory diagnostic examinations in faecal samples preventively								
Yes	No			Yes	No			
5	61			17	196			0.91
Total visits made annually by veterinarians to the farm during the preceding season								
0 – 4	5 – 7	> 7		0 – 4	5 – 7	> 7		
29	16	21		94	56	63		0.92
Application of reproductive control practices in the farm								
Yes	No			Yes	No			
13	53			64	149			0.10
Nutritional modifications before the lambing period								
Yes	No			Yes	No			
43	23			150	63			0.42
Age of lamb removal from their dams								
≤ 40 days	41 – 60 days	> 60 days		≤ 40 days	41 – 60 days	> 60 days		
21	37	8		78	111	24		0.78
Seasonal transfer of animals to other site								
Yes	No			Yes	No			
11	55			36	177			0.96
Annual frequency of systemic disinfections in the farm								
0 – 9	> 9			0 – 9	> 9			
60	6			191	22			0.80
Shearing of animals								
Yes	No			Yes	No			
65	1			210	3			0.95
Provision of finished feed (concentrate) to animals throughout the year								
Yes	No			Yes	No			
62	4			198	15			0.78
Age of farmers								
Up to 50 years	Over 50 years			Up to 50 years	Over 50 years			
37	29			129	84			0.51

Length of previous animal farming experience of the farmer						
≤ 5 years	> 5 years		≤ 5 years	> 5 years		
14	52		42	171		0.79
Education of the farmer						
Primary education	Secondary or post-secondary education	Tertiary education	Primary education	Secondary or post-secondary education	Tertiary education	
16	42	8	30	153	30	0.15
Professional involvement in farming						
Full-time	Part-time		Full-time	Part-time		
62	4		187	26		0.16
Presence of working staff in the flock						
Yes	No		Yes	No		
24	42		78	135		0.97

Table S9. Associations of high proportion (> 63%) of *Teladorsagia* spp. among Trichostrongylidae helminths in faecal samples from 279 sheep flocks in Greece, as found in univariable analysis.

Number of farms								*p*
**Farms with high proprotion (> 63%) of *Teladorsagia* spp. among Trichostorngylidae helminths in faecal samples (*n* = 94)**				**Farms with low proprotion (≤ 63%) of *Teladorsagia* spp. among Trichostorngylidae helminths in faecal samples (*n* = 185)**				
Management system applied in the farm								
Intensive	Semi-intensive	Semi-extensive	Extensive	Intensive	Semi-intensive	Semi-extensive	Extensive	
8	42	34	10	28	86	58	13	0.31
Years at the present location								
0 – 2	3 – 5	> 5		0 – 2	3 – 5	> 5		
2	8	84		3	22	160		0.67
Month into the lactation period at sampling								
0–1st	2nd–5th	6th–9th	After 9th	0–1st	2nd–5th	6th–9th	After 9th	
1	43	45	5	4	74	96	11	0.76
Availability of a main building for animals								
Yes	No			Yes	No			
93	1			179	6			0.27
Material of the floor of the barn								
Soil	Other			Soil	Other			
82	12			170	15			0.21
Availability of straw bedding								
Yes	No			Yes	No			
70	24			157	28			0.035
Annual frequency of removal / clean-up of the straw bedding								
1 – 2	> 2			1 – 2	> 2			
46	24			101	56			0.84
Grazing practiced								
Yes	No			Yes	No			
86	8			156	29			0.10
Grazing land available to animals								
0 – 0.5 acre per animal	> 0.5 acre per animal			0 – 0.5 acre per animal	> 0.5 acre per animal			
29	65			69	116			0.29
No. of female animals in the farm								
0 – 165	166 – 330	331 – 500	> 500	0 – 165	166 – 330	331 – 500	> 500	
24	35	21	14	51	61	39	34	0.82
Breed of ewes								
Cross-breeds	Greek breeds	Imported breeds		Cross-breeds	Greek breeds	Imported breeds		
18	41	35		21	81	83		0.17

Month of the start of the lambing season								
All year	Autumn	Winter	Spring-Summer	All year	Autumn	Winter	Spring-Summer	
5	70	17	2	10	140	32	3	0.99
Collaboration with a veterinarian								
Yes	No	Yes	No					
76	18	158	27					0.33
Use of laboratory diagnostic examinations in faecal samples preventively								
Yes	No	Yes	No					
6	88	16	169					0.51
Total visits made annually by veterinarians to the farm during the preceding season								
0 – 4	5 – 7	> 7	0 – 4	5 – 7	> 7			
42	29	23	81	43	61			0.23
Application of reproductive control practices in the farm								
Yes	No	Yes	No					
28	67	49	136					0.60
Nutritional modifications before the lambing period								
Yes	No	Yes	No					
59	35	134	51					0.10
Age of lamb removal from their dams								
≤ 40 days	41 – 60 days	> 60 days	≤ 40 days	41 – 60 days	> 60 days			
27	56	11	72	92	21			0.23
Seasonal transfer of animals to other site								
Yes	No	Yes	No					
19	94	28	157					0.70
Annual frequency of systemic disinfections in the farm								
0 – 9	> 9	0 – 9	> 9					
86	8	165	20					0.55
Shearing of animals								
Yes	No	Yes	No					
94	0	181	4					0.15
Provision of finished feed (concentrate) to animals throughout the year								
Yes	No	Yes	No					
85	9	175	10					0.19
Age of farmers								
Up to 50 years	Over 50 years	Up to 50 years	Over 50 years					
65	29	102	83					0.024

Length of previous animal farming experience of the farmer				
≤ 5 years	> 5 years	≤ 5 years	> 5 years	
17	77	39	146	0.55

Education of the farmer						
Primary education	Secondary or post-secondary education	Tertiary education	Primary education	Secondary or post-secondary education	Tertiary education	
18	65	11	28	130	27	0.61

Professional involvement in farming				
Full-time	Part-time	Full-time	Part-time	
83	11	166	19	0.72

Presence of working staff in the flock				
Yes	No	Yes	No	
30	64	72	113	0.25

Table S10. Associations of high proportion (> 29%) of *Haemonchus contortus* among Trichostrongylidae helminths in faecal samples from 279 sheep flocks in Greece, as found in univariable analysis.

**Number of farms**								***p***
**Farms with high proprotion (> 29%) of *H. contortus* among Trichostorngylidae helminths in faecal samples (*n* = 160)**				**Farms with low proprotion (≤ 29%) of *H. contortus* among Trichostorngylidae helminths in faecal samples (*n* = 119)**				
Management system applied in the farm								
Intensive	Semi-intensive	Semi-extensive	Extensive	Intensive	Semi-intensive	Semi-extensive	Extensive	
24	78	47	11	12	50	45	12	0.23
Years at the present location								
0 – 2	3 – 5	> 5		0 – 2	3 – 5	> 5		
3	18	139		2	12	105		0.94
Month into the lactation period at sampling								
0–1st	2nd–5th	6th–9th	After 9th	0–1st	2nd–5th	6th–9th	After 9th	
4	58	89	9	1	59	52	7	0.12
Availability of a main building for animals								
Yes	No			Yes	No			
154	6			118	1			0.12
Material of the floor of the barn								
Soil	Other			Soil	Other			
145	15			107	12			0.84
Availability of straw bedding								
Yes	No			Yes	No			
136	24			91	28			0.070
Annual frequency of removal / clean-up of the straw bedding								
1 – 2	> 2			1 – 2	> 2			
86	50			61	30			0.56
Grazing practiced								
Yes	No			Yes	No			
134	26			108	11			0.09
Grazing land available to animals								
0 – 0.5 acre per animal	> 0.5 acre per animal			0 – 0.5 acre per animal	> 0.5 acre per animal			
62	98			36	83			0.14
No. of female animals in the farm								
0 – 165	166 – 330	331 – 500	> 500	0 – 165	166 – 330	331 – 500	> 500	
47	47	36	30	28	49	24	18	0.23
Breed of ewes								
Cross-breeds	Greek breeds	Imported breeds		Cross-breeds	Greek breeds	Imported breeds		
17	76	67		22	46	51		0.12

Month of the start of the lambing season								
All year	Autumn	Winter	Spring-Summer	All year	Autumn	Winter	Spring-Summer	
9	116	32	3	6	94	17	2	0.64
Collaboration with a veterinarian								
Yes	No	Yes	No					
137	23	97	22					0.36
Use of laboratory diagnostic examinations in faecal samples preventively								
Yes	No	Yes	No					
10	150	12	107					0.24
Total visits made annually by veterinarians to the farm during the preceding season								
0 – 4	5 – 7	> 7	0 – 4	5 – 7	> 7			
70	37	53	53	35	31			0.33
Application of reproductive control practices in the farm								
Yes	No	Yes	No					
46	114	31	88					0.62
Nutritional modifications before the lambing period								
Yes	No	Yes	No					
116	44	77	42					0.16
Age of lamb removal from their dams								
≤ 40 days	41 – 60 days	> 60 days	≤ 40 days	41 – 60 days	> 60 days			
60	81	19	39	67	13			0.64
Seasonal transfer of animals to other site								
Yes	No	Yes	No					
21	139	26	93					0.05
Annual frequency of systemic disinfections in the farm								
0 – 9	> 9	0 – 9	> 9					
141	19	110	9					0.24
Shearing of animals								
Yes	No	Yes	No					
157	3	118	1					0.47
Provision of finished feed (concentrate) to animals throughout the year								
Yes	No	Yes	No					
149	11	111	8					0.96
Age of farmers								
Up to 50 years	Over 50 years	Up to 50 years	Over 50 years					
88	72	78	41					0.08

Length of previous animal farming experience of the farmer				
≤ 5 years	> 5 years	≤ 5 years	> 5 years	
35	125	21	98	0.38

Education of the farmer						
Primary education	Secondary or post-secondary education	Tertiary education	Primary education	Secondary or post-secondary education	Tertiary education	
27	114	19	19	81	19	0.62

Professional involvement in farming				
Full-time	Part-time	Full-time	Part-time	
146	14	103	16	0.21

Presence of working staff in the flock				
Yes	No	Yes	No	
63	97	39	80	0.26

Table S11. Associations of presence of *Trichuris* spp. in faecal samples from 279 sheep flocks in Greece, as found in univariable analysis.

		Number of farms		p
		**Farms with presence of *Trichuris* spp. in faecal samples (n = 61)**	**Farms with no presence of *Trichuris* spp. in faecal samples (n = 218)**	
Management system applied in the farm	Intensive	7	29	0.48
	Semi-intensive	27	102	
	Semi-extensive	19	72	
	Extensive	8	15	
Years at the present location	0 – 2	1	3	0.52
	3 – 5	9	21	
	> 5	51	193	
Month into the lactation period at sampling	0–1st	2	3	0.78
	2nd–5th	25	92	
	6th–9th	31	110	
	After 9th	3	13	
Availability of a main building for animals	Yes	60	212	0.62
	No	1	6	
Material of the floor of the barn	Soil	53	199	0.30
	Other	8	19	
Availability of straw bedding	Yes	48	179	0.54
	No	13	39	
Annual frequency of removal / clean-up of the straw bedding	1 – 2	32	115	0.76
	> 2	16	64	
Grazing practiced	Yes	54	188	0.64
	No	7	30	
Grazing land available to animals	0 – 0.5 acre per animal	25	73	0.28
	> 0.5 acre per animal	36	145	
No. of female animals in the farm	0 – 165	24	50	0.005
	166 – 330	10	87	
	331 – 500	15	45	
	> 500	12	36	
Breed of ewes	Cross-breeds	11	28	0.52
	Greek breeds	27	95	
	Imported breeds	23	95	

Month of the start of the lambing season								
All year	Autumn	Winter	Spring-Summer	All year	Autumn	Winter	Spring-Summer	
8	42	11	0	7	168	38	5	0.015
Collaboration with a veterinarian								
Yes	No			Yes	No			
54	7			180	38			0.26
Use of laboratory diagnostic examinations in faecal samples preventively								
Yes	No			Yes	No			
4	57			18	200			0.66
Total visits made annually by veterinarians to the farm during the preceding season								
0 – 4	5 – 7	> 7		0 – 4	5 – 7	> 7		
20	19	22		103	53	62		0.13
Application of reproductive control practices in the farm								
Yes	No			Yes	No			
19	42			58	160			0.48
Nutritional modifications before the lambing period								
Yes	No			Yes	No			
42	19			151	67			0.95
Age of lamb removal from their dams								
≤ 40 days	41 – 60 days	> 60 days		≤ 40 days	41 – 60 days	> 60 days		
19	35	7		80	113	25		0.71
Seasonal transfer of animals to other site								
Yes	No			Yes	No			
11	50			36	182			0.78
Annual frequency of systemic disinfections in the farm								
0 – 9	> 9			0 – 9	> 9			
52	9			199	19			0.17
Shearing of animals								
Yes	No			Yes	No			
61	0			214	4			0.29
Provision of finished feed (concentrate) to animals throughout the year								
Yes	No			Yes	No			
54	7			206	12			0.10
Age of farmers								
Up to 50 years	Over 50 years			Up to 50 years	Over 50 years			
43	18			123	95			0.048

Length of previous animal farming experience of the farmer						
≤ 5 years	> 5 years	≤ 5 years	> 5 years			
14	47	42	176			0.53
Education of the farmer						
Primary education	Secondary or post-secondary education	Tertiary education	Primary education	Secondary or post-secondary education	Tertiary education	
15	38	8	31	157	30	0.15
Professional involvement in farming						
Full-time	Part-time	Full-time	Part-time			
54	7	195	23			0.84
Presence of working staff in the flock						
Yes	No	Yes	No			
26	35	76	142			0.27

Table S12. Associations of presence of lungworms in faecal samples from 279 sheep flocks in Greece, as found in univariable analysis.

Number of farms								***p***
**Farms with presence of lungworms in faecal samples (*n* = 54)**				**Farms with no presence of lungworms in faecal samples (*n* = 225)**				
Management system applied in the farm								
Intensive	Semi-intensive	Semi-extensive	Extensive	Intensive	Semi-intensive	Semi-extensive	Extensive	
6	26	16	6	30	102	76	17	0.77
Years at the present location								
0 – 2	3 – 5	> 5		0 – 2	3 – 5	> 5		
1	6	47		4	24	197		0.99
Month into the lactation period at sampling								
0–1st	2nd–5th	6th–9th	After 9th	0–1st	2nd–5th	6th–9th	After 9th	
1	24	25	4	4	93	116	12	0.88
Availability of a main building for animals								
Yes	No			Yes	No			
54	0			218	7			0.19
Material of the floor of the barn								
Soil	Other			Soil	Other			
50	4			203	22			0.59
Availability of straw bedding								
Yes	No			Yes	No			
44	10			183	42			0.98
Annual frequency of removal / clean-up of the straw bedding								
1 – 2	> 2			1 – 2	> 2			
32	12			115	68			0.22
Grazing practiced								
Yes	No			Yes	No			
48	6			194	31			0.60
Grazing land available to animals								
0 – 0.5 acre per animal	> 0.5 acre per animal			0 – 0.5 acre per animal	> 0.5 acre per animal			
21	33			77	148			0.52
No. of female animals in the farm								
0 – 165	166 – 330	331 – 500	> 500	0 – 165	166 – 330	331 – 500	> 500	
16	22	11	5	59	74	49	43	0.33
Breed of ewes								
Cross-breeds	Greek breeds	Imported breeds		Cross-breeds	Greek breeds	Imported breeds		
7	25	22		32	97	96		0.91

Month of the start of the lambing season								
All year	Autumn	Winter	Spring-Summer	All year	Autumn	Winter	Spring-Summer	
3	38	12	1	12	172	37	4	0.79
Collaboration with a veterinarian								
Yes	No			Yes	No			
44	10			190	35			0.59
Use of laboratory diagnostic examinations in faecal samples preventively								
Yes	No			Yes	No			
2	52			20	205			0.21
Total visits made annually by veterinarians to the farm during the preceding season								
0 – 4	5 – 7	> 7		0 – 4	5 – 7	> 7		
20	18	16		103	54	68		0.33
Application of reproductive control practices in the farm								
Yes	No			Yes	No			
16	38			61	164			0.71
Nutritional modifications before the lambing period								
Yes	No			Yes	No			
38	16			155	70			0.83
Age of lamb removal from their dams								
≤ 40 days	41 – 60 days	> 60 days		≤ 40 days	41 – 60 days	> 60 days		
18	31	5		81	117	27		0.74
Seasonal transfer of animals to other site								
Yes	No			Yes	No			
8	46			39	186			0.66
Annual frequency of systemic disinfections in the farm								
0 – 9	> 9			0 – 9	> 9			
49	5			202	23			0.83
Shearing of animals								
Yes	No			Yes	No			
52	2			223	2			0.12
Provision of finished feed (concentrate) to animals throughout the year								
Yes	No			Yes	No			
48	6			212	13			0.16
Age of farmers								
Up to 50 years	Over 50 years			Up to 50 years	Over 50 years			
34	20			132	93			0.56

Length of previous animal farming experience of the farmer						
≤ 5 years	> 5 years		≤ 5 years	> 5 years		
12	42		44	181		0.66
Education of the farmer						
Primary education	Secondary or post-secondary education	Tertiary education	Primary education	Secondary or post-secondary education	Tertiary education	
9	39	6	37	156	32	0.83
Professional involvement in farming						
Full-time	Part-time		Full-time	Part-time		
51	3		198	27		0.17
Presence of working staff in the flock						
Yes	No		Yes	No		
19	35		83	142		0.82
